# Supplementary material for: Structure of the transcription activator target Tra1 within the chromatin modifying complex SAGA
Source: Nat Commun. 2017 Nov 16;8:1556. doi: 10.1038/s41467-017-01564-7 (PMC5691046; doi:10.1038/s41467-017-01564-7)
Supplement: Supplementary file 1 — Supplementary Information [file 41467_2017_1564_MOESM1_ESM.pdf]

Supplementary Table 1: Cryo-EM data collection and processing summary.

Microscope	FEI Titan Krios
Voltage, kV	300
Detector	Falcon II
Defocus range, um	1.4 - 3.4
Exposure time, s	1
Frames	7
Total dose (fluence) per movie, e/A[2]	60
Dose (fluence) per frame, e/A[2]	7
Microscope sessions	4
Movies[a]	8505
Pixel size, A/px	1.1
Box size, px	512
Raw particles[b]	264901
Final particles[c]	105916
Final resolution (full complex)	11.7
Final resolution (lobe A)	5.7

a - number of movies used for processing
b - number of particles after auto-picking
c - number of particles used in final reconstruction

a

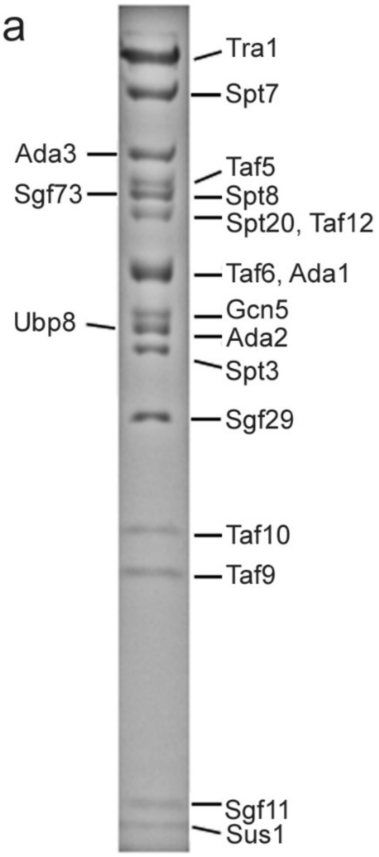

b

Tra1	1.00
Spt7	1.09
Taf5	1.37
Ada2	1.79
Spt8	1.66
Taf6	1.44
Spt20	1.42
Sgf73	0.88
Ada3	0.90
Gcn5	1.00
Sgf29	1.79
Ubp8	0.79
Ada1	0.99
Taf12	0.50
Taf10	1.06
Taf9	0.99
Sus1	0.93
Spt3	0.50
Sgf11	0.51

Supplementary Figure 1 | Purification of endogenous SAGA complex from P.pastoris. (a) Colloidal coomassie blue stained SDS-PAGE analysis of the subunit composition of SAGA complexes purified from the SBP-tagged Sgf73 strain. The identity of all SAGA subunits bands is indicated. **(b)** Proteomic analysis of the purified SAGA complexes. The spectral abundance factors (SAFs) values of each protein were averaged over 4 experiments and normalized to the SAF value of Tra1.

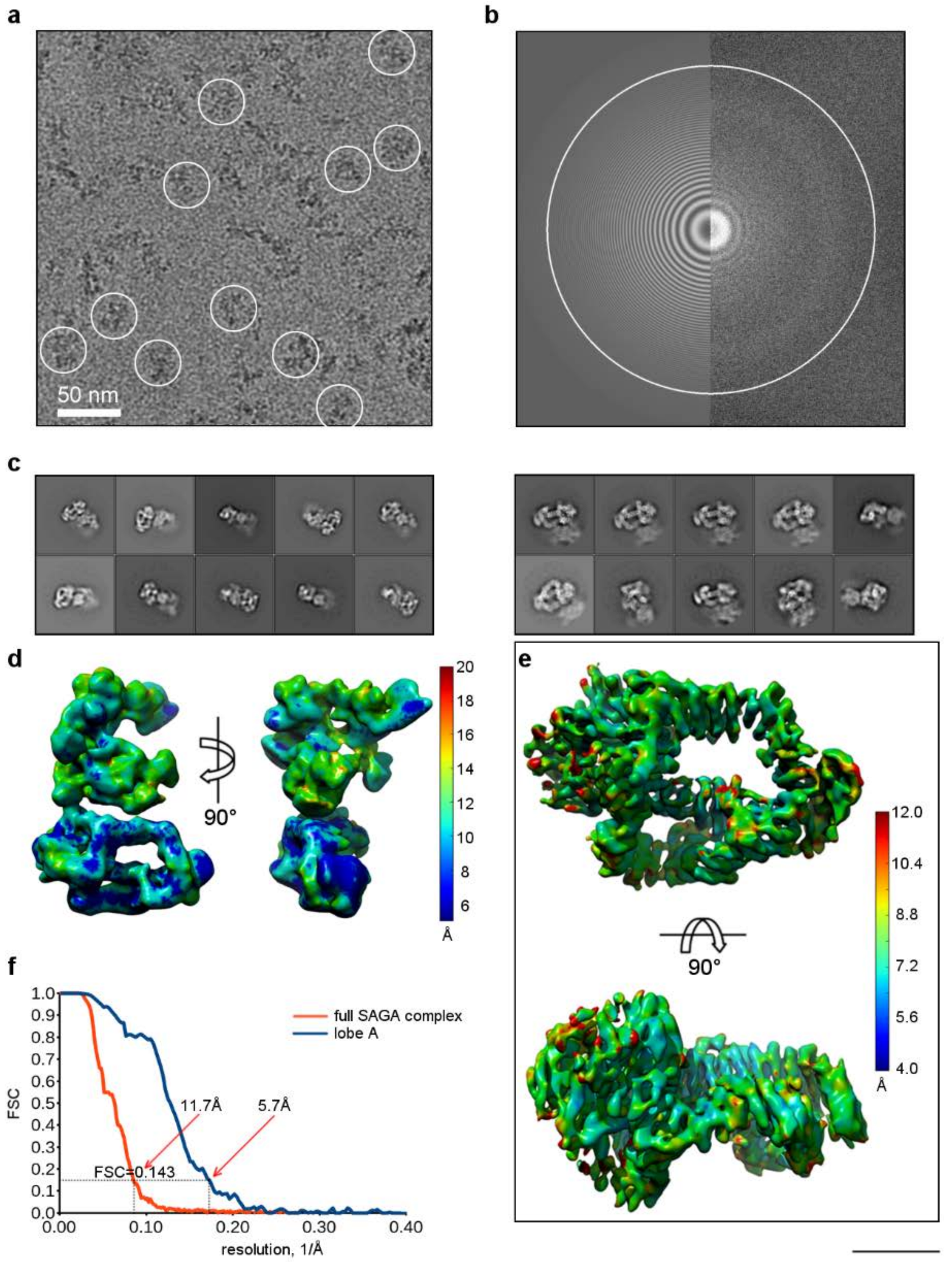

Supplementary Figure 2 | Single-particle cryo-EM analysis of SAGA.

(a) Original images of frozen-hydrated PpSAGA complexes. **(b)** Power spectrum of the Fourier transform from a characteristic image (right) and estimated Contrast Transfer Function (left). **(c)** Two-dimensional class averages obtained using the relion software for the entire SAGA complex (left) or after localized refinement on lobe A (right). **(d)** Local resolution map obtained for the entire SAGA molecules using the Resmap software. **(e)** Local resolution map of lobe A using the Resmap software. **(f)** Fourier Shell Correlation curve as a function of spatial frequencies in 1/Å for the entire SAGA complex (Red) and lobe A (blue). Scale bar is 10 nm in (d) and 5.4 nm in (e).

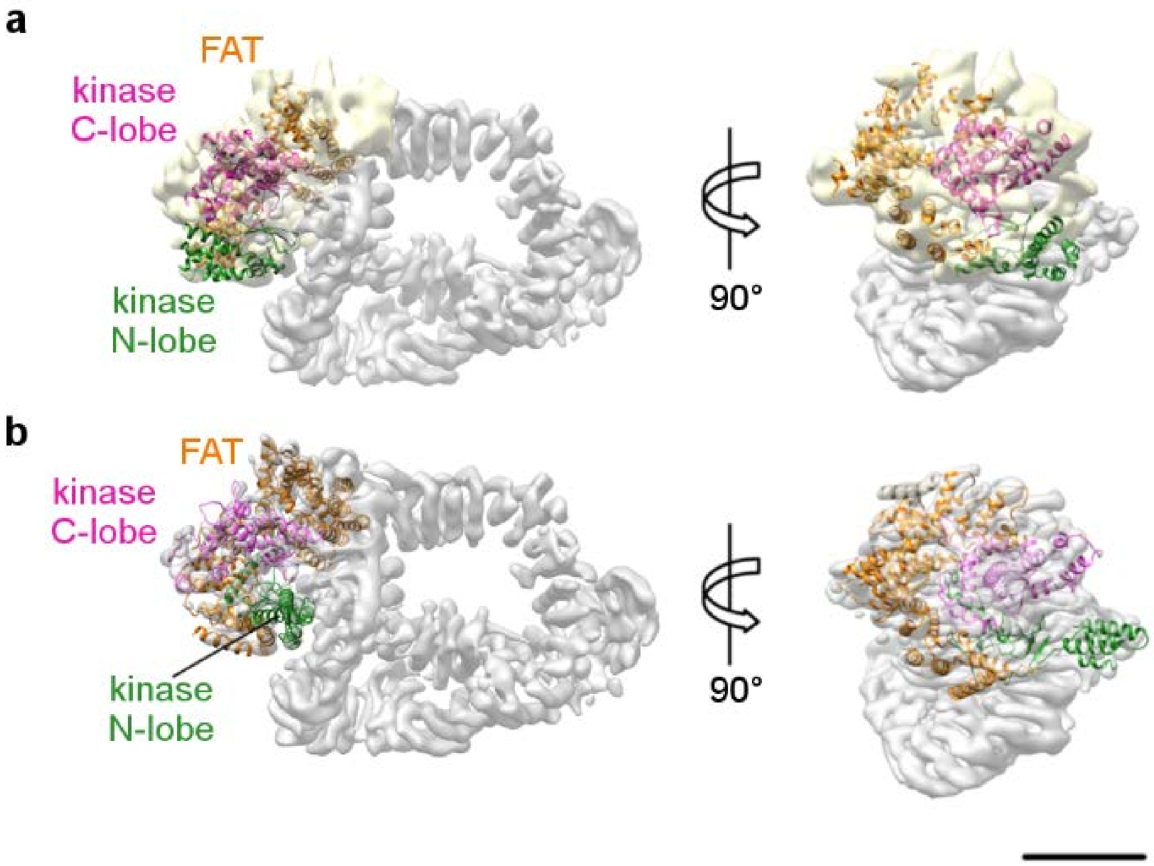

Supplementary Figure 3 | Fitting of the FAT-kinase module.

(a) Docking of the mTOR FAT and Kinase crystal structures into the Tra1 cryoEM envelope. **(b)** Position of the corresponding domains after homology modelling and flexible fitting into the cryo-EM densities.

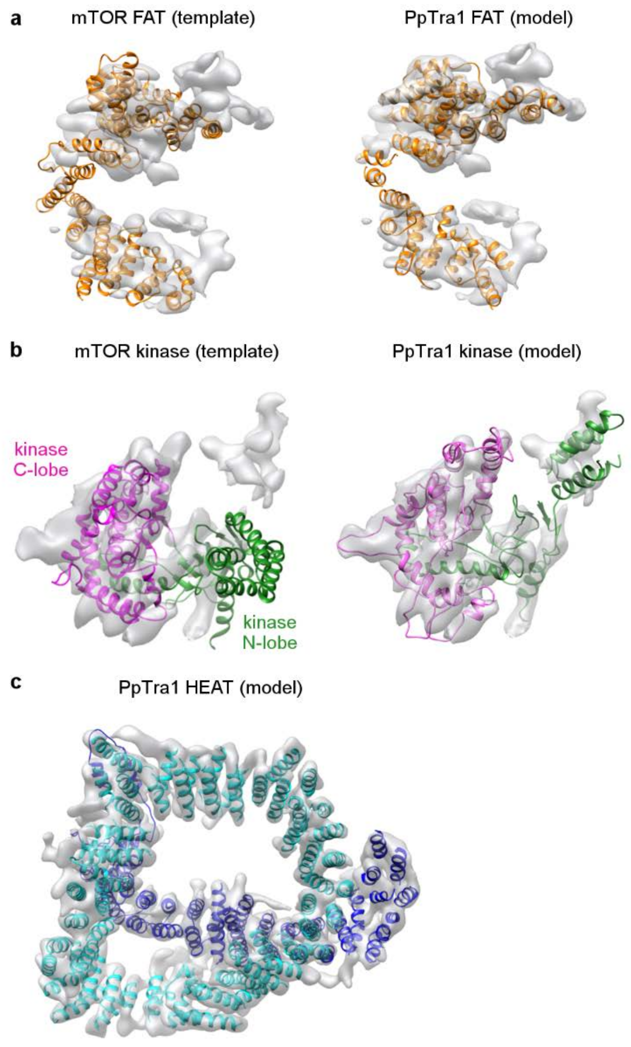

Supplementary Figure 4 | Fit of the atomic models into the cryoEM density.

(a) Fit of the mTOR FAT domain into the cryo-EM envelope before (left) and after (right) homology modelling and flexible fitting **(b)**. Fit of the mTOR kinase domain into the cryo-EM envelope before (left) and after (right) homology modelling and flexible fitting **(c)** Modelling of the HEAt repeats into the cryo-EM envelope

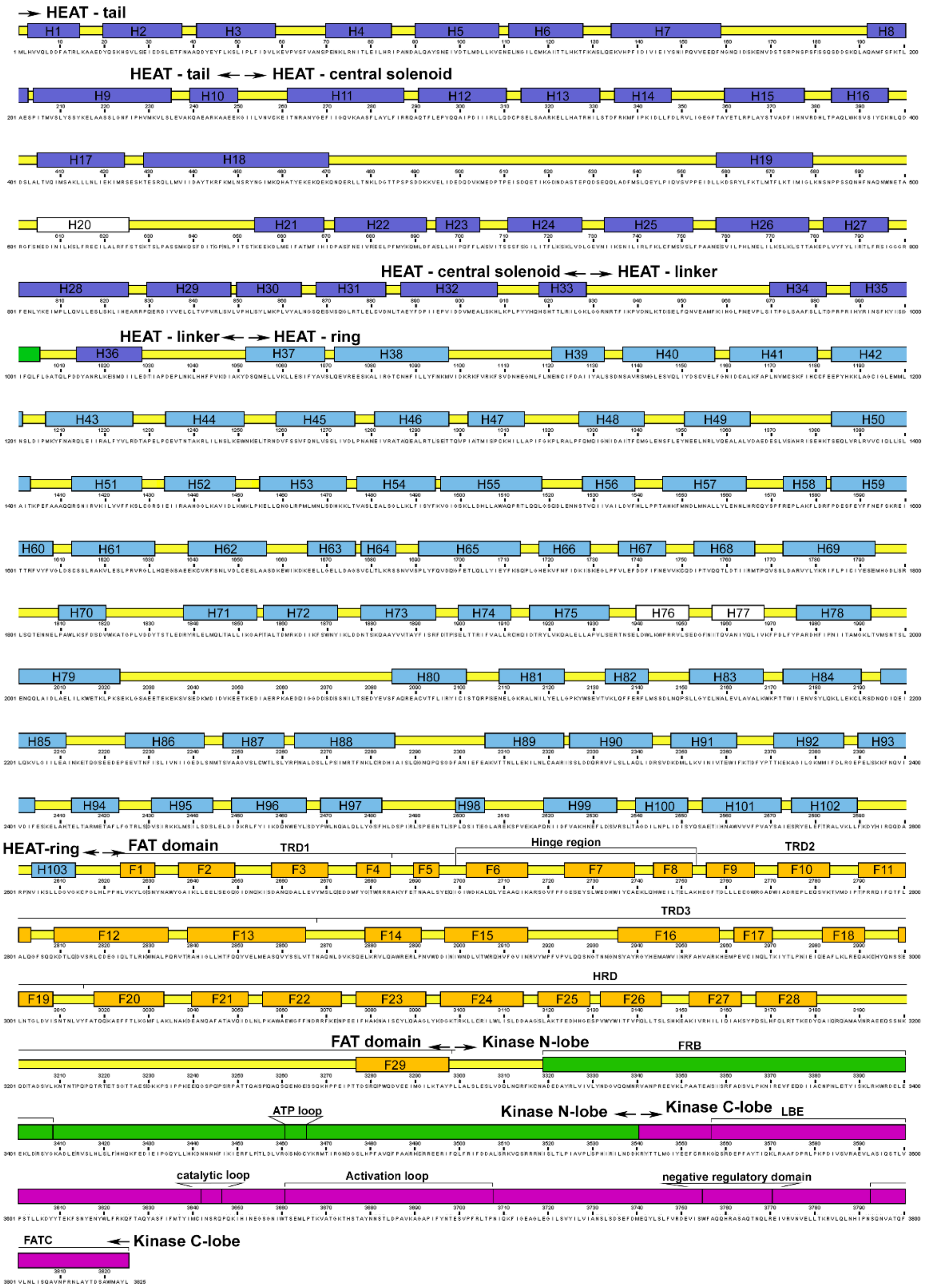

Supplementary Figure 5 | Secondary structure assignment of the Tra1 cryo-EM map: Diagram showing the secondary structure prediction and assignment of the *P. pastoris* Tra1 subunit within the cryo EM map. Helices are numbered according to secondary structure prediction from H1 to H103 for the HEAT domain and from F1 to F29 for the FAT domain. The predicted helices H20, H76 and H77 were not found in the electron density and may correspond to wrong predictions or to helices placed within loops outside of the α-solenoid structure.

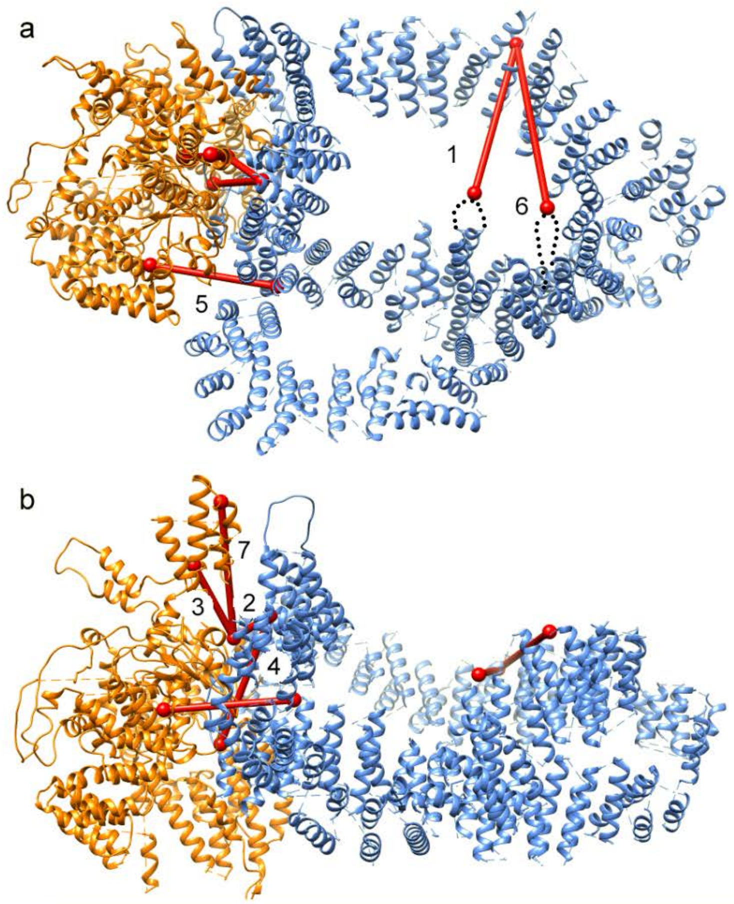

c

XL number	*S. Cerevisiae*	*P. Pastoris*		*Length (Å)*
1	512 - 2351	481 (in loop)	2353	45
2	1059 - 1232	1039 (in loop)	1212	15
3	1059 - 3324	1039	3402	24
4	1232 - 1051	1027 (in loop)	1212	24
5	1340 - 3175	1320 (in loop)	3177	34
6	1971 - 2351	1947 (in loop)	2353	56
7	3304 - 1059	3382	1039	40

Supplementary Figure 6 | Position of cross-linked residues: (a) and **(b)** show two orthogonal views of Tra1 in which chemically cross-linked residues reported in[13] are represented by red dots connected by red lines. Residues 481 and 1947 are placed within loops represented by dotted lines. **(c)** Table showing the 7 reported intra-subunit cross-links as identified in S. cerevisiae, with their corresponding coordinates in P. pastoris and the distance measured according to the structure. Several cross-linked residues are

placed within loops which may explain that the measured distance may be longer than the length of the cross-linking agent (30 Å).

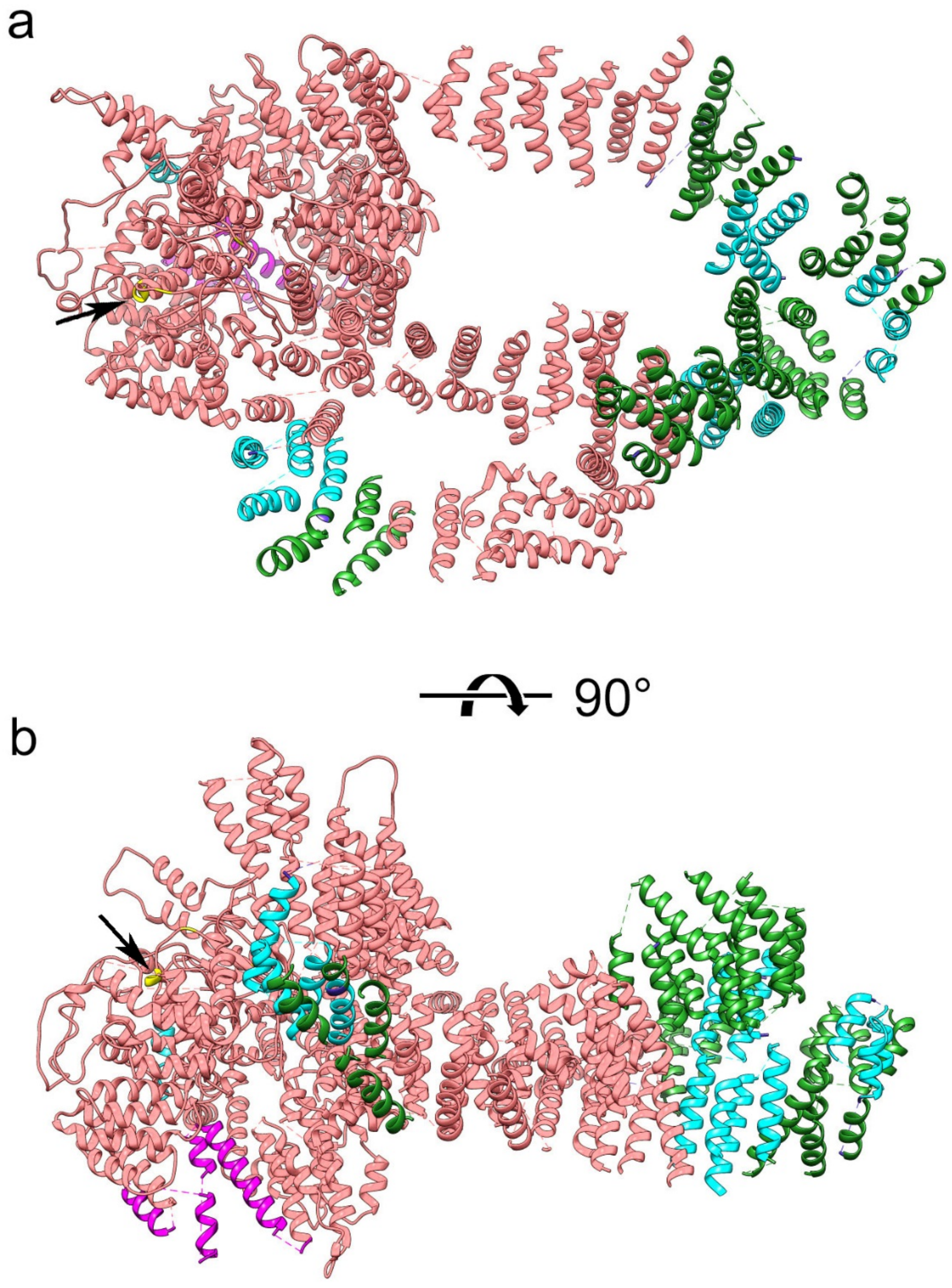

Supplementary Figure 7 | Mapping of Tra1 mutants with functional defects. Tra1 deletion mutants, from 60 to 100 residues in size, reported by Knutson and Hahn[23] were mapped on the P. pastoris Tra1 structure. Inviable mutants which probably affect the stability or the integration of Tra1 into SAGA are represented in light red, viable mutants with little phenotypes are colored green, mutants that affect promoter recruitment are

shown in cyan, mutants showing acetylation defects are colored magenta. A triple mutant $SRR_{3413}AAA$, giving rise to decreased relative amounts of acetylated histone H3 and histone H4[24], is shown in yellow and highlighted by an arrow.
